# Giant cystic lymphangioma of the mesentery: varied clinical presentation of 3 cases

**Published:** 2012-05-12

**Authors:** Mohamed Rami, Abdelhalim Mahmoudi, Aziz El Madi, Khalid, Khattala, Moulay Abderrahmane Afifi, Youssef Bouabdallah

**Affiliations:** 1 Department of pediatric surgery, CHU Hassan II, Fez, Morocco

**Keywords:** Lymphangioma, mesentery, cyst

## Abstract

Giant cystic lymphangioma is an uncommon mesenteric tumor which is usually reported in children. In this paper, we describe 3 cases, that was admitted in our department, two with abdominal distension, pain, and an abdominopelvic mass; the other present an acute abdomen. Preoperative studies including abdominal ultrasonography and computed tomography failed to determine the cause of the pain for the female patients. The laparotomy found a giant cystic tumor of the small bowel mesentery. The histological study showed a tumor that was diagnosed as a cystic lymphangioma. Based on those three cases a review of the literature is suggested.

## Introduction

Cystic lymphangioma is an uncommon mesenteric tumor which is usually reported in children, it is a benign, slow growing tumor derived from of the lymphatic vessels; it is rarely found as intra-abdominal masses when occurring in the abdomen [1–3]. The abdominal ultrasonography, the CT-scan and celioscopy might be useful for establishing the diagnosis [1, 5]. The treatment is mainly surgical; it consists of enucleation when feasible; the segmental intestinal resection is achieved when the cyst adheres intimately to the bowel [3, 6]. We describe 3 cases of giant cystic lymphangioma with variant clinical presentation, treated surgically, with very good result.

## Patient and case report

### Observation 1

**Figure 1:**
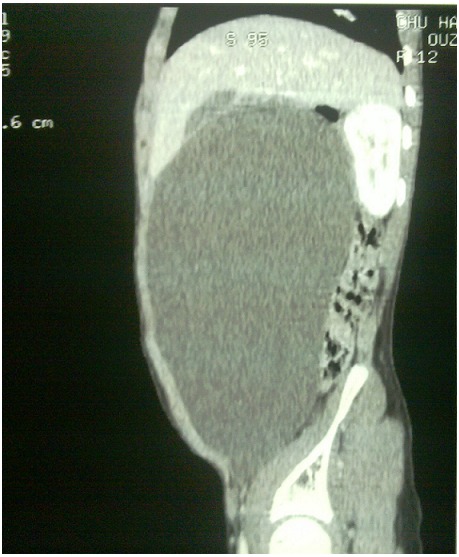
Sagittal view of the abdominal CT scan of the third patient, demonstrating a huge cystic mass

**Figure 2:**
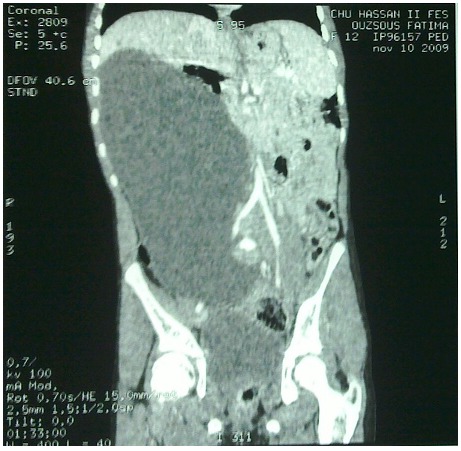
Abdominal CT scan of the same patient, coronal view

**Figure 3:**
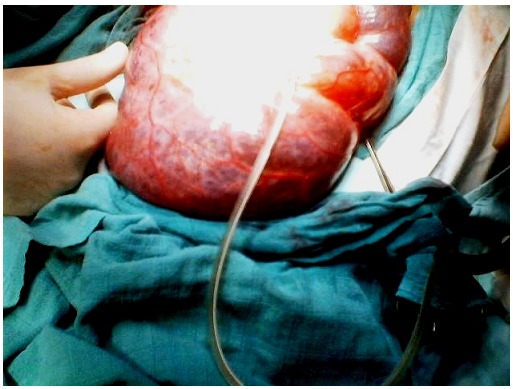
Giant mesenteric tumor was discovered using laparotomy in the first patient; it contained 5 liters of clear fluid

**Figure 4:**
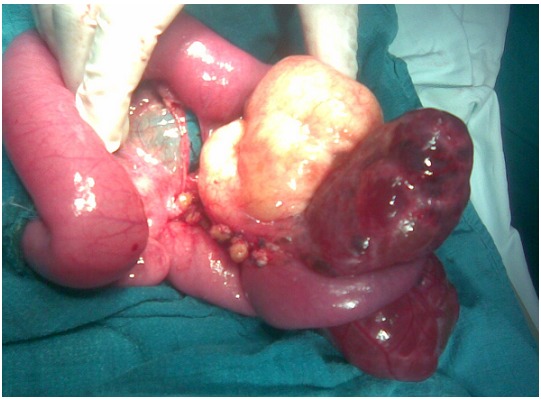
A cystic mass of the small bowel mesentery that contains a serosanguinous fluid represents cyst hemorrhage in the second child

A 12 year old female was referred to our hospital for a clinical profile characterized by nausea, vomiting and abdominal ballottement during eight previous days. This child has had a history of constipation, and the intermittent medical treatment administered for 2 years has failed. The examination has found a cachectic child with a very swollen abdomen, and a palpable abdominopelvic mass. The biological assessments investigated the blood cell count, the alpha-fetoprotein and the beta human chorionic gonadotrophin, and were found normal. The full abdominal X-ray showed a displaced bowel loop by the mass of soft tissue (Figure 1). The ultrasonography revealed an abdominal multiloculated septated cystic mass measuring 20 cm. The CT-scan demonstrated a septated cystic mass associated to a dilatation of the right kidney (Figure 2), without visualization of the ovaries. The surgery was performed, with the diagnosis of an ovarian tumor, and a huge mesenteric tumor containing 5 litters of clear fluid was discovered (Figure 3), the involved bowel and the mesenteric tumor were resected (Figure 4). A primary anastomosis was performed, and the diagnosis of the cystic lymphangioma was revealed by the histological study of the surgical piece. The surgical follow was simple, and the clinical evolution was very good.

### Observation 2

**Figure 5:**
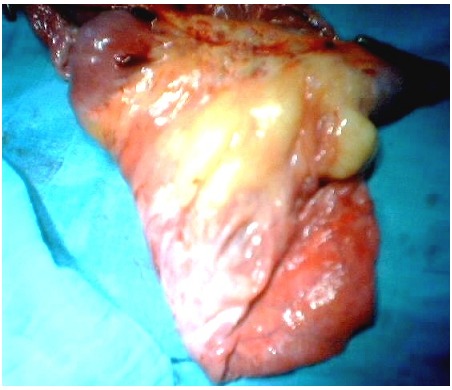
The resected surgical piece, first patient

**Figure 6:**
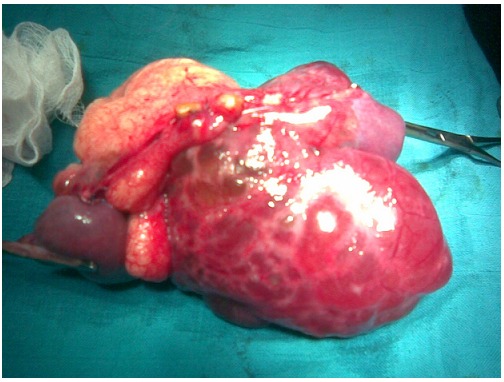
The tumor and the involved bowel resected, in the second patient

A 10 years old boy was admitted in the emergency for an acute abdomen in the last two days, with fever. The physical examination showed a very painful abdomen, with tenderness and muscle guarding in the left side of the abdomen. The blood cell count found a hyperleucocytosis 21000 E/mm^3^. The ultrasonography revealed an abdominal multiloculated septated cystic mass measuring 14 cm. The CT-scan demonstrated a septated cystic mass of 17×11×4 cm. The patient was underwent surgery. A cystic mass of the small bowel mesentery, containing a serosanguinous fluid representing cyst hemorrhage (Figure 5) was discovered. The tumor and the involved bowel were resected (Figure 6), and an anastomosis was performed. The histological study confirms the diagnosis of lymphangioma. The clinical evolution was good.

### Observation 3

An 11 year old girl was admitted to our department for abdominal pain, and increase volume during 7 months. She also has constipation. The examination has found a cachectic child with a palpable abdomino-pelvic mass. The biological assessments investigated the blood cell count, the alpha-fetoprotein and the beta human chorionic gonadotrophin, and were found normal. The full abdominal X-ray showed a displaced bowel loop by the mass of soft tissue. The ultrasonography revealed an abdominal cystic mass measuring 20 cm. The CT-scan demonstrated a cystic mass measuring 25/15/10, without visualization of the ovaries. The surgery was performed, and a huge mesenteric tumor containing 4 litters of clear fluid was discovered, the involved bowel and the mesenteric tumor were resected. A primary anastomosis was performed, and the diagnosis of the cystic lymphangioma was revealed by the histological study of the surgical piece. The surgical follow was simple, and the clinical evolution is simple within of 2 years recession.

## Discussion

Cystic cavernous lymphangiomas are uncommon benign slow growing tumor derived from the lymphatic vessels [1]. It etiology is unknown and might be associated with developmental anomalies of the lymphatics [2]. It is more frequently found in childhood (60% are present at birth, and 90% are detected by the end of the second year), and mostly diagnosed in the first five years of life [1, 4]. Occasionally, the tumor is also discovered in adult in various other anatomic sites [1]. It most often occurs in the head and neck, axillae, or groin of young children. It is rarely found as intra-abdominal masses when occurring in the abdomen (2-10% in the internal organs) [2, 3]. Cystic lymphangiomas might arise with acute abdominal pain associated to bowel obstruction (like the second patient), signs of peritonitis [2–4], chronic abdominal swelling that is detected by palpation of a cystic mass or abdominal swollenness with lower extremities lymphoedema [5–7]. The radiology is the revealing diagnostic tool; the abdominal ultrasonography is the procedure of choice for establishing the diagnosis, even during the antenatal stage [1, 8]. The acute lymphangiomas associating intracystic hemorrhage are more difficult to diagnose, CT-scan and celioscopy might be useful approaches in this context [8]. Sequential ultrasonography and CT-scan examinations showed progressive enlargement of the cystic masses, increase of fluid echogenicity and wall thickening associating multiplication of septa [9]. In our study the differential diagnosis with an ovarian tumor, was difficult, for the female patients.

The final diagnosis is always based on the histological findings, since this examination shows extensive myofibroblastic areas and objectifies the lymphatic character of the lesion [4, 6].

Where the underlying lesion could be discerned, the tumor was composed of cystically dilated lymphatic spaces, which were partially invested by a layer of smooth muscle and were associated to with occasional lymphoid aggregates. The lymphatic spaces contained either clear fluid or large numbers of foamy macrophages. The lymphatic endothelial cells lining the cystic spaces were generally attenuated without any cytological atypism [4, 7]. The immunohistochemistry showed that the endothelial cells lining the dilated lymphatic spaces were positive for CD31, D2-40, CD34, and all were negative for keratin [4, 10]. The treatment is mainly surgical; it consists of enucleation when feasible; the segmental intestinal resection is achieved when the cyst adheres intimately to the bowel [3, 6, 9]. Few reported cases of diffuse malformation required an extensive bowel resection, which might yield short bowel syndrome [3, 9]. The resection could be performed with laparoscopic technique without large incisions [2, 4]. Tumors were cystic masses associated to areas of fat necrosis and hemorrhage. Often, cysts contain thick, gelatinous or milky fluid [7]. The sclerosis techniques constitute an interesting alternative and complementary treatment approach, intracystic sclerotherapy using doxycycline is possible for unresectable lymphangiomas [7, 10]. The local recurrence of the tumor is possible [10].

## Conclusion

The cystic mesenteric lymphangioma is rare benign tumor. The clinical symptomatology is polymorphic and not specific. The diagnosis is suggested by the imaging modalities, but still requiring an histopathologic confirmation. The treatment of choice is surgical and consists of a full resection of the lesion. The intracystic sclerotherapy could be used for symptomatic tumors associating diffuse mesenteric lesion which are not resecable without extensive intestinal sacrifice.
